# Matrix Gla protein regulates adipogenesis and is serum marker of visceral adiposity

**DOI:** 10.1080/21623945.2020.1721692

**Published:** 2020-01-31

**Authors:** Chaomin Li, Jing Li, Fang He, Kun Li, Xu Li, Yan Zhang

**Affiliations:** a Center for Translational Medicine, The First Affiliated Hospital of Xi’an Jiaotong University, Xi’an, Shaanxi, People’s Republic of China; b Honghui Hospital, Xi’an Jiaotong University, Xi’an, Shaanxi, People’s Republic of China; c Clinical Laboratory, ShanXi Mineral Hospital, Xi’an, Shaanxi, People’s Republic of China

**Keywords:** MGP, obesity, dp-ucMGP, adipocyte

## Abstract

**Objective** Matrix Gla protein (MGP) is a potent calcification inhibitor. *Mgp*-/- mice display increased proportion of brown adipose tissue. However, whether MGP is involved in fat metabolism remains unclear. This study aims to investigate the involvement. **Methods** Expression of adipocyte differentiation markers was examined by RT-qPCR. Adipocyte formation was assessed by Oil Red staining. Serum triglyceride, cholesterol, and desphosphorylated-uncarboxylated MGP (dp-ucMGP) were quantified by ELISA. Visceral fat was detected by bioelectrical impedance analysis. **Results** MGP is highly expressed in visceral fat. MGP expression is induced during preadipocyte differentiation. Knockout of MGP leads to retardation of 3T3-L1 differentiation. Intracellular triglyceride amount is impaired while glycerol release is increased in MGP-depleted cells. Serum dp-ucMGP level is significantly increased in individual with higher visceral fat index (VFI) and waist height ratio (WHtR), but not body mass index (BMI). Additionally, dp-ucMGP positively correlates to low-density lipoprotein cholesterol (LDL-C) level. **Conclusions** MGP is involved in fat metabolism and serum inactive MGP level is associated with visceral fat. Our study uncovers for the first time the link between MGP and fat metabolism, and sheds light on the potential of dp-ucMGP as a novel serum marker.

## Introduction

Obesity, in which excess fat accumulates in the body, is one of the most prevalent public health problems nowadays. In 2015, 600 million adults (12%) and 100 million children were obese worldwide [1]. Obesity increases the incidence of various diseases, including cardiovascular diseases, type 2 diabetes, osteoarthritis, depression, and cancer [2,3]. The cause of obesity is a combination of excessive food intake, lack of physical activity, and genetic susceptibility [4]. Traditionally, people with body mass index (BMI, weight/the square of the height) higher than 30 kg/m^2^ is considered to be obese. However, increasing evidence shows that BMI could not reflect overweight or obesity accurately, since body weight could be gained from other high-density tissues such as muscle. In addition, BMI could not distinguish the fat distribution in the whole body, which has distinct contribution to health [5].

Adipose tissue distributes to various location in the body, leading to various type of fat such as visceral fat inside the abdominal cavity, and subcutaneous fat underneath the skin [6]. It is increasingly clear that different types of body fat have distinct functions. Visceral adipose buffers dietary lipids by storing excess calories in the form of triglycerides [7]. The characteristic that high expression and activity of β3-adrenoreceptors and fewer insulin receptor makes visceral adipose more capable of metabolizing lipids than other fat types [7]. Subcutaneous adipose provides insulation, cushioning, and serves as a long-term energy storage depot [8]. Usually, subcutaneous fat is not harmful for health, while excess visceral fat is linked to obesity-related diseases, such as type 2 diabetes, insulin resistance, and inflammatory diseases [9,10].

When energy intake exceeds expenditure, adipose tissue expands through forming new adipocytes (hyperplasia) or enlargement of existing adipocytes (hypertrophy). These physiological processes are necessary metabolic adjustments for adequate energy storage. Adipocyte is the main component and functional unit of adipose tissue. It is originated from mesenchymal stem cells (MSCs) which differentiate into various cell types in response to specific environmental cues [11]. The differentiation of adipocyte requires the concerted regulation of expression of adipogenic genes, which is controlled by the key transcriptional factor peroxisome proliferator activated receptor γ (PPARγ) [11]. Adipocytes store lipid and secrete various metabolic adipokines, playing essential roles in the regulation of metabolic homoeostasis [12]. Dysregulation of adipogenesis is associated with impaired ability to store excess lipids, leading to hypertrophic and insulin-resistant adipocytes [13]. For instance, the adipocyte differentiate is reduced in high-risk individuals who have relatives with type 2 diabetes [13].

Matrix Gla protein (MGP) is a small 14 kDa vitamin K-dependent protein. It is well known as a potent calcification inhibitor, involved in vascular calcium metabolism [14]. Fully activation of MGP requires post-translational modifications including γ-carboxylation and phosphorylation. Different states of modification leads to existence of various MGP isoforms, such as phosphorylated (pMGP), carboxylated (cMGP), uncarboxylated (ucMGP), desphosphorylated (dpMGP), and desphosphorylated-uncarboxylated MGP (dp-ucMGP) [15]. Dp-ucMGP has low affinity for vascular calcium, and circulating dp-ucMGP is reported to be associated with increased risk for atherosclerosis and cardiovascular disease (CVD) [16,17].

Mutch and colleagues demonstrated that MGP is dramatically increased during preadipocyte differentiation in microarray. MGP protein is highly secreted from adipocytes, acting as a novel adipokine [18,19]. Recently, Lanham and colleagues reported that the proportion of brown adipose tissue to body fat is increased in *Mgp* knockout mice [20]. These above findings prompted us to investigate the role of MGP in fat metabolism. In the present study, we find that MGP is highly expressed in fat, especially the visceral fat in mice. We investigated the function of MGP in adipogenesis, and detected the association of serum inactive MGP with adiposity. Our results show that MGP regulates differentiation, lipid storage, and lipolysis of 3T3-L1 cells. Serum dp-ucMGP level is positive associated with visceral adiposity. Our study uncovers the link between MGP function and fat metabolism, sheds light on the potential of dp-ucMGP as a novel serum marker.

## Materials and method

### Cell culture and induction

Mouse 3T3-L1 preadipocytes (ATCC, Manassas, VA, USA) were grown in complete Dulbecco’s modified Eagle’s medium (DMEM, Invitrogen, Carlsbad, CA, USA) supplemented with 10% calf blood serum (CBS) and 1% penicillin/streptomycin at 37°C, 5% CO_2_ and 95% humidity. For the induced differentiation assay, 2 days after reaching confluence, 3T3-L1 were induced with DMEM containing 10% foetal bovine serum (FBS) plus 1 μM dexamethasone, 1 μg/mL insulin, 0.5 mM methylisobutylxanthine (IBMX) for 48 h. Then the media was replaced with DMEM containing 10% foetal bovine serum (FBS) plus 1 μg/mL insulin every 2 days. Cells were harvested at the indicated time points for gene expression analyses.

### CRISPR/Cas9-mediated MGP gene knockout

Control single guide RNA (sgRNA) or sgRNA targeting exon 1 of mouse Mgp gene were synthesized, respectively. The oligonucleotides were annealed and ligated into the LentiCRISPRv2 vector (Addgene 52961). Then 293T cells at 70% confluence were transfected with 1 μg of recombinant backbone vector, 750 ng of psPAX2 (Addgene 12260), and 250 ng of pVSVg (Addgene 31947). Twelve hours after transfection, the medium was changed. After an additional 36 h, the culture medium was harvested. Once 3T3-L1 cells reached 50% confluence, they were incubated with virus-containing medium for 48 h and further selected in medium containing 2 μg/ml of puromycin for an additional 3 days before validation of the knockout efficiency and the following experiments.

### Oil Red O staining

Cells were fixed with 10% formalin for 1 h and stained with 0.5% Oil Red O solution for 60 min. After washing with PBS, the cells were photographed. Then Oil Red O retained in the cells was eluted with 100% isopropanol and the absorbance was measured at 490 nm. The adipocyte size was measured using Image J.

### Reverse transcription quantitative real-time PCR (RT-qPCR)

Total RNA was extracted using TRIzol (Invitrogen) according to the manufacturer’s instructions. Two  microgram of RNA was reverse-transcribed into first-strand cDNA using the GoldScript one-step RT-PCR Kit (TaKaRa, Japan). For qPCR analysis, each sample contained 200 nM of primer, 20 ng of cDNA and 2X SYBR Green PCR Master Mix (TaKaRa) to make up a total reaction volume of 20 μL. qPCR analyses were performed on the Bio-Rad CFX96 Real-Time PCR System. Primer sequences of the primers are as follows:

5′-AAGAGAGTCCAGGAACGCAA −3′ (sense) and 5′-GGTTGTAGGCAGCGTTGTAG-3′ (antisense) for MGP, 5ʹ-GGAGATCTCCAGTGATATCGACCA-3ʹ (sense) and

5ʹ-ACGGCTTCTACGGATCGAAACT-3ʹ (antisense) for PPARγ,

5ʹ-TGGACAAGAACAGCAACGAC-3ʹ (sense) and

5ʹ-TCACTGGTCAACTCCAGCAC-3ʹ (antisense) for C/EBPα,

5ʹ-AAGAAGTGGGAGTGGGCTTT-3ʹ (sense) and

5ʹ-ATGATCATGTTGGGCTTGGC-3ʹ (antisense) for FABP4,

5ʹ-TACAACCAACAGAATCATTATGACGG-3ʹ (sense) and

5ʹ-GAAAGCCAGTAAATAGAGTCGTTGA-3ʹ (antisense) for adiponectin, and 5′-TTCGACAGTCAGCCGCATCTTCTT-3′ (sense) and 5′-CAGGCGCCCAATACGACCAAATC-3′ (antisense) for GAPDH. The comparative Ct (2^ − ΔΔCt^) method was used to obtain mean mRNA values normalized by GAPDH. All experiments were carried out with at least three repeats.

### Western blotting

Cells were harvested and lysed in radioimmunoprecipitation assay (RIPA) buffer containing protease inhibitor mixture (Roche, Branchburg, NJ, USA). Protein concentrations were measured using BCA protein assay (Pierce, Rockford, IL, USA). The cell lysates were resolved by 10% sodium dodecylsulfate-polyacrylamide gel electrophoresis (SDS-PAGE) and transferred to PVDF membranes. After blocking in 0.1% casein in PBS, membranes were incubated with primary antibodies (anti-MGP, Abcam Cambridge, MA, USA, ab86233, 1:500) and corresponding peroxidase-conjugated secondary antibodies (Jackson Immuno Research, West Grove, PA, USA). Signals were developed using Hyglo chemiluminescent reagent (Invitrogen) and detected using a ChemiDoc MP (Bio-Rad, Hercules, CA, USA).

### Triglyceride and glycerol assay

3T3-L1 cells were induced for 8 days. After being washed with PBS, cells were harvested and sonicated for 3 min. Then the suspension was centrifuged at 3500 rpm for 10 min. Intracellular triglycerides were quantified using a triglyceride kit (Sigma, St. Louis, MO, USA) according to the manufacturer’s instructions. Cells were induced for 8 days, then cells were treated with 10 μM isoproterenol for 6 h followed by quantification of glycerol release using a kit (EnzyChrom™ Glycerol Assay Kit, BioAssay Systems, USA).

### Animal treatment

Two-month-old C57/Bl6 mice were maintained at 22 ± 2°C with a regular light-dark cycle (12-h light and 12-h dark) and had free access to food and water. All animal manipulations were approved by the Ethical Committee of the Xi’an Jiaotong University. The mice were random divided into two groups, control group feeding with regular diet and water, and warfarin treated group feeding with water containing 300 ng/mL warfarin (Sigma). The body weight was measured every week. After 8 weeks of treatment, the serum was collected for assaying the triglyceride and cholesterol. Body fat rate (BFR, fat weight/total body weight) was obtained using a Dual X-ray Digital Imagining System (Medikors, South Korea). Adipose tissue was collected and fixed with 10% neutral buffered formalin, followed by paraffin embedding and Oil Red O staining.

### Clinical samples

The cross-sectional study was conducted in the southern area of Shaanxi Province in China, in November 2018. This study has been approved by Ethical Committee of Xi’an Jiaotong University. A randomly selected sample of 278 subjects aged 35 years or more were included in the study. Participates were divided into different groups according to their VFI, WHtR, or BMI value. According to the Working Group on Obesity in China (WGOC) criteria [21], a BMI of 18.5 kg/m^2^ − 23.9 kg/m^2^ as normal weight, of 24 kg/m^2^ − 27.9 kg/m^2^ as overweight, and of 28.0 kg/m^2^ or higher as obesity. Central obesity was defined as WHtR ≥0.5. VFIs of 1–4.5 are considered to be healthy, whereas 5–9.5 to be need alert, 10–14.5 to be obese, and more than 15 to be dangerous. Standard measurements for weight and VFI were detected by bioelectrical impedance analysis using Omron body composition monitor (V-body HBF-371, OMRON, Kyoto, Japan). Height was measured with a standard stadiometer. BMI was calculated as weight/height^2^. BMI and VFI were employed to evaluate the overall obesity and visceral fat condition, respectively. Waist circumstance was measured to the nearest 0.1 cm, midway between the lowest rib and the superior border of the iliac rest, with a flexible anthropometeric tape. WHtR was calculated as waist circumference/height. Participants were kept in fasting condition before the serum measurement. LDL-Cand HDL-C were detected using automatic biochemical metre (DXC-800, Beckman). After centrifuge, supernatants of blood were collected. An ELISA kit was used to detect the dp-ucMGP level (ImmunoDiagnosticSystems, IDS, Tyne & Wear, UK).

### Statistical analysis

All data are representative of at least three independent experiments. Data are expressed as mean ± SEM unless noted otherwise. Correlations between visceral fat parameters and dp-ucMGP were determined using Spearman’s correlation. All the other experiments were analysed using 2-tailed unpaired Student’s t-test for two groups by Prism (GraphPad Software). *P*< 0.05 was considered significant.

## Results

### MGP is involved in adipocyte differentiation

**Figure 1.**
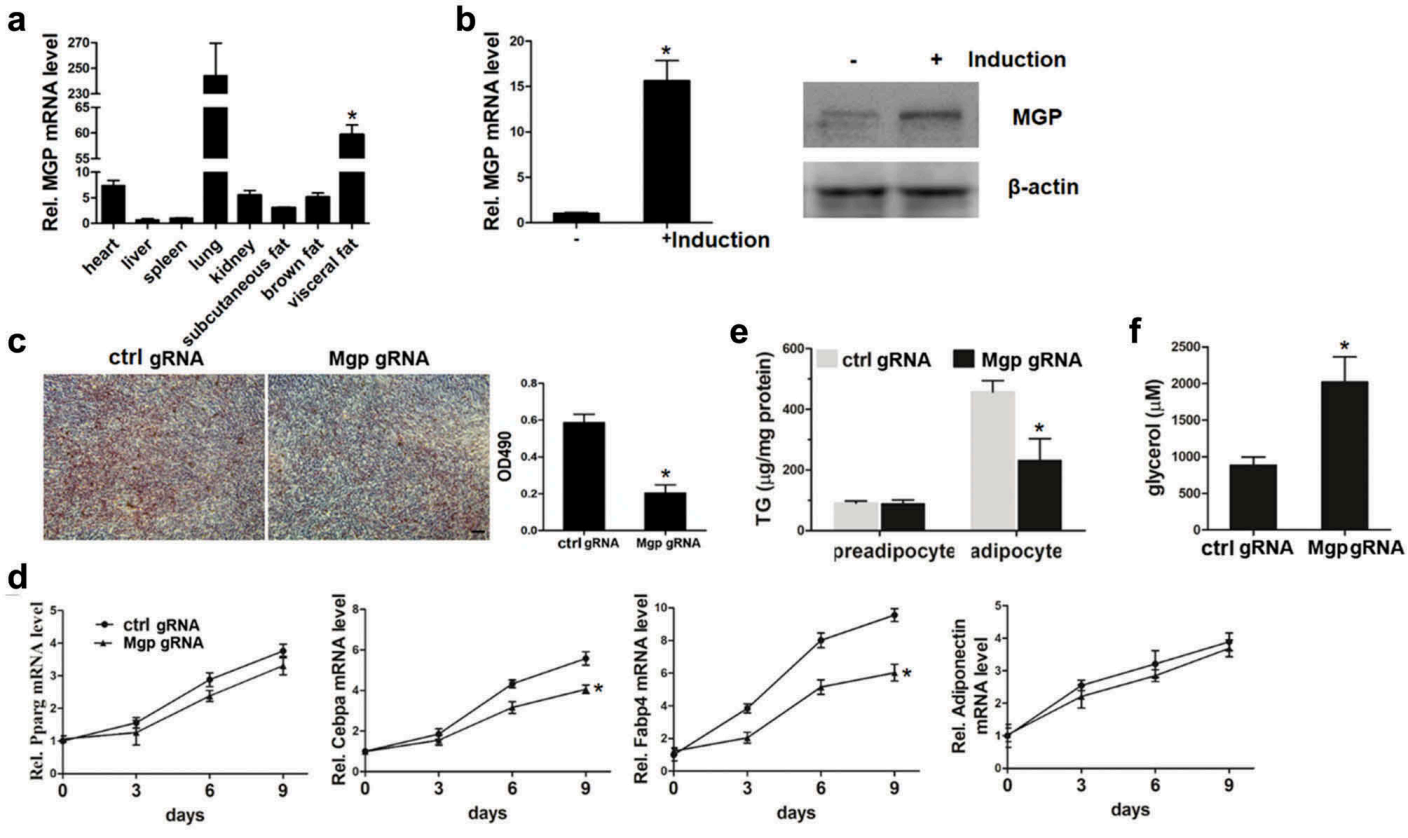
MGP is involved in adipocyte differentiation

We detected the expression level of MGP in heart, liver, spleen, lung, kidney, and fat tissues including subcutaneous, visceral, and brown fat. We found that consistent with the previous report, MGP is significantly higher in lung. Compared to the other tissues (liver, spleen, *etc.*), its expression in fat tissue, especially in visceral fat tissue is significantly higher (Figure 1(a)). Until recently there is no report, to our knowledge, regarding the involvement of MGP in fat. This prompted us to investigate the role of MGP in fat metabolism. We employed the classical adipocyte differentiation model 3T3-L1 cells, to examine whether MGP has effects on adipogenesis. Preadipocytes 3T3-L1 cells were induced with adipogenetic agents, and MGP mRNA before and after induction was determined. As shown in Figure 1(b), MGP mRNA and protein expression is significantly induced after 3T3-L1 differentiation. Then we knocked out the *Mgp* gene in the 3T3-L1 cells using lentivirus expressing CRISPR-Cas9 and targeted sgRNA. Modified and control 3T3-L1 cells were induced into adipocytes, and Oil Red staining was performed to examine the differentiation of 3T3-L1. As shown in Figure 1(c), the number of mature adipocyte (Oil Red positive) is less in the MGP deficient cells. The mRNA levels of adipogenetic markers including PPARγ, CEBPα, FABP4, and adiponectin were also examined. Showed by the qPCR data, the expression of CEBPα and FABP4 are decreased, however, expression of key transcriptional factor PPARγ and adiponection remain unchanged in the absence of MGP (Figure 1(d)). These results prompted us to clarify whether the decreased number of mature adipocytes in MGP deficient cells is caused by inhibition of adipogenesis or stimulation of lipolysis. We detected the accumulation and release of intracellular lipid in the absence of MGP. As shown in Figure 1(e), the accumulation of intracellular triglycerides is impaired in differentiated MGP deficient cells. The release of glycerol, an indicator of lipolysis, is significantly elevated in MGP knockout cells (Figure 1(f)). These results demonstrated that MGP functions as a dual regulator in both adipogenesis and lipolysis.

(a) MGP expression in different tissues. Tissues from 4-weeks old male C57/Bl6 mice were collected, and mRNA levels of MGP were determined by RT-qPCR. The MGP mRNA values were normalized by GAPDH. n = 3; (b) MGP expression is increased after adipocyte differentiation. 3T3-L1 cells were induced for 8 d, and cells were harvested for MGP mRNA and protein analysis. The MGP mRNA values were normalized by GAPDH. n = 3; (c) Lipid accumulation is decreased in MGP deficient cells. 3T3-L1 cells were induced for 8 d and Oil Red staining was performed. Scale Bar = 100 μm; (d) Effects of MGP knockout on expression of adipogenetic markers. 3T3-L1 cells were induced for the indicated time points and mRNA analysis was performed to determine the mRNA expression of adipogenetic markers. The mRNA values were normalized by GAPDH. n = 3; (e) The accumulation of lipid is decreased in MGP knockout cells. After induction for 8 d, cells were harvested for intracellular triglycerides assay. n = 5; (f) The release of glycerol is increased in MGP deficient cells. 3T3-L1 cells were induced for 8 d, and the glycerol release was determined. n = 5.

### Warfarin treatment leads to change of fat metabolism in mice

**Figure 2.**
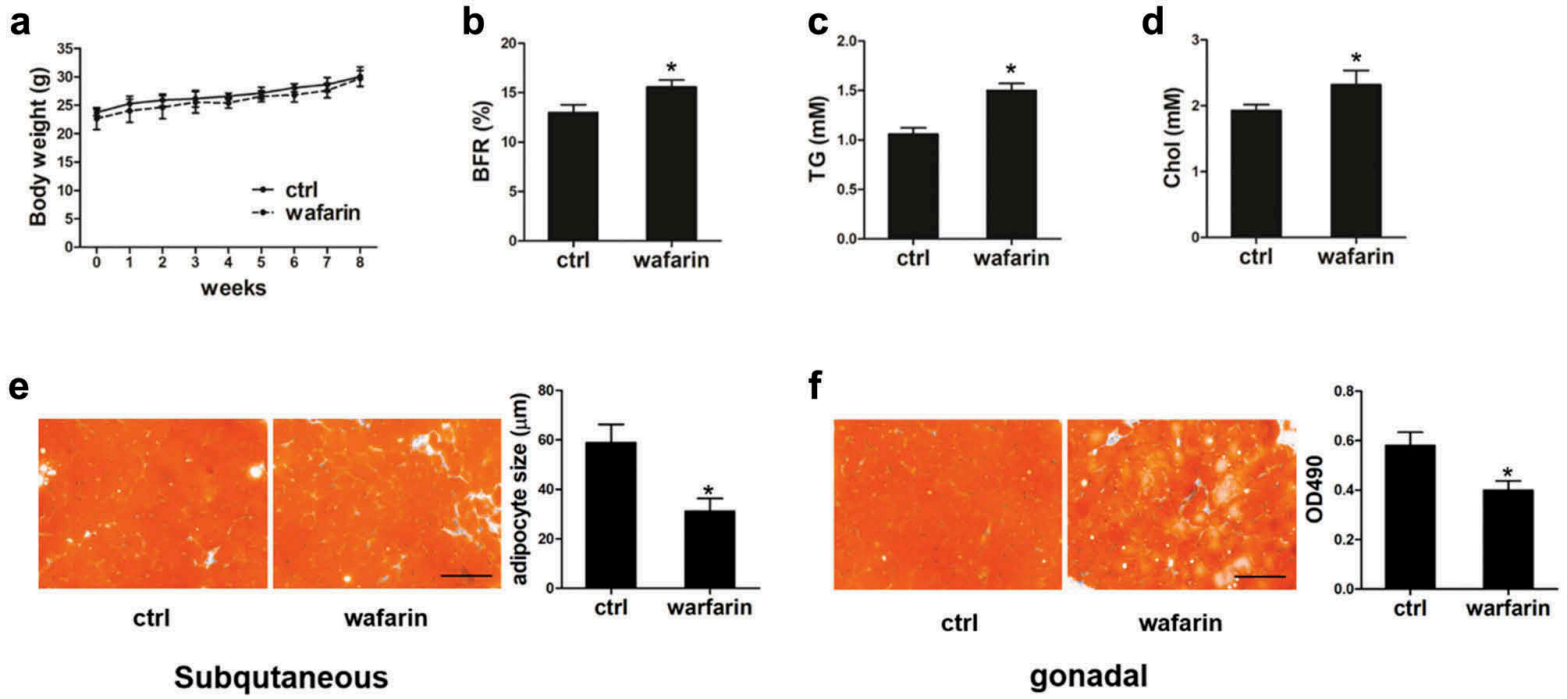
Effects of warfarin on release and accumulation of lipid

To determine the effects of MGP *in vivo*, we used warfarin which is widely used as MGP inhibitor in research [22–26], to block the function of MGP and examined its role *in vivo*. C57/Bl6 mice were feeding with water or 300 ng/mL warfarin for 8 weeks. As shown in Figure 2(a), the body weight is comparable between control and treated mice. However, the body fat rate (BFR) is significantly increased in warfarin-treated mice after 8 weeks of treatment (Figure 2(b)). The other DEXA indicators were shown in Figure S1. The serum level of the triglyceride (TG) and cholesterol (Chol) was quantified. Both are significantly increased in the warfarin treated mice (Figure 2(c) and (d)). Adipose tissue was collected and stained with Oil Red O. As shown in Figure 2(e), although the overall positive Oil Red O signal are similar between the two groups, the size of adipocyte in the subcutaneous fat tissues of warfarin-treated mice is much smaller. As for the gonadal fat tissue, the lipid accumulation is decreased due to the warfarin treatment (Figure 2(f)). These results demonstrated that MGP regulates fat metabolism *in vivo*.

(a) Effects of warfarin on mice body weight. Mice was treated with 300 ng/mL warfarin for 8 weeks, and body weight was measured. n = 6; (b) Body fat rate (BFR) is increased after warfarin treatment. After 8 weeks of treatment, BFR was measured by DEXA. n = 6; (c) and (d) Serum lipid is increased after warfarin treatment. Serum was collected for the quantification of triglyceride (c) and glycerol (d) after 8 weeks of treatment. n = 6; (e) Adipocyte size of subcutaneous fat is smaller after warfarin treatment. Subcutaneous fat was fixed and stained with oil Red; (f) Lipid accumulation in gonadal fat is decreased in warfarin treated mice. Gonadal fat was fixed and stained with oil Red. Scale Bar = 100 μm.

### Serum dp-ucMGP is increased in individuals with higher VFI or WHtR

**Figure 3.**
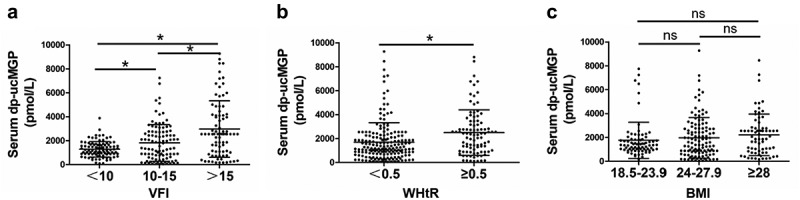
Dp-ucMGP is increased in individuals with higher VFI or WHtR

Fully activation of MGP requires post-translational modifications including γ-carboxylation and phosphorylation. Therefore, serum desphosphorylated-uncarboxylated (dp-ucMGP) level could reflect the status of MGP function *in vivo*. Given the essential function of MGP in fat metabolism, we analysed the association of serum dp-ucMGP level and obesity in human. A cohort of random 278 participants were included in this study. The characteristics of these participants are summarized in Table 1. The mean age of the study population is 46.5 years. There are 74 (26.6%) participants with hypertension (HT), 52 (18.7%) with coronary artery disease CAD, and 49 (17.6%) with diabetes mellitus (DM). The average value of serum glucose, cholesterol, TG, HDL-C, LDL-C is 7.31, 6.43, 1.81, 1.52, 2.61 mmol/L, respectively (Table 1). We used this cohort and compared the levels of serum dp-ucMGP of individuals with different obesity related indicators, including visceral fat index (VFI) which is often ignored by people and reflects abdominal obesity. As shown in Figure 3(a), the dp-ucMGP level is significantly elevated with the increase of VFI, indicating a positive correlation between serum dp-ucMGP and VFI. Waist height ratio (WHtR) is another index reflecting the visceral fat amount. We found that serum dp-ucMGP level is significantly elevated in the group with higher WHtR compared to the group with lower WHtR (Figure 3(b)). However, indicated by Figure 3(c), there is no difference in the dp-ucMGP levels between different body mass index (BMI) groups. These results demonstrated that serum dp-ucMGP level is associated with abdominal obesity.

**Table 1.** General data of the 278 participants included in this analysis

Parameter	Value (mean±SD)
Age, years	46.5 ± 11.1
Gender, M/F	136/142
BMI	25.4 ± 3.7
*Medical history*	
HT, n (%)	74 (26.6%)
CAD, n (%)	52 (18.7%)
DM, n (%)	49 (17.6%)
Other chronic disease	18 (6.5%)
*Biochemical measurements*	
Glucose, mmol/L	7.31 ± 0.72
Cholesterol, mmol/L	6.43 ± 2.33
TG, mmol/L	1.81 ± 1.25
HDL-C, mmol/L	1.52 ± 0.40
LDL-C, mmol/L	2.61 ± 0.99
dp-ucMGP, pmol/L	1974.56 ± 1759.43

(a) Serum dp-ucMGP levels in different VFI groups. Participants were divided by the visceral fat index (VFI), and serum dp-ucMGP level was determined. n = 96 for VFI<10, n = 99 for 10≤ VFI≤15, n = 83 for VFI>15; (b) Serum dp-ucMGP levels in different WHtR groups. Participants were divided by the waist height ratio (WHtR), and serum dp-ucMGP level was compared in the two groups. n = 183 for WHtR<0.5, n = 94 for WHtR≥0.5, respectively; (c) Serum dp-ucMGP levels in different BMI groups. Participants were divided by the body mass index (BMI), and serum dp-ucMGP level was compared between groups. n = 77 for 18.5≤ BMI≤23.9, n = 116 for 24≤ BMI≤27.9, n = 69 for BMI≥28, respectively.

### Serum dp-ucMGP positively correlated with LDL-C

**Figure 4.**
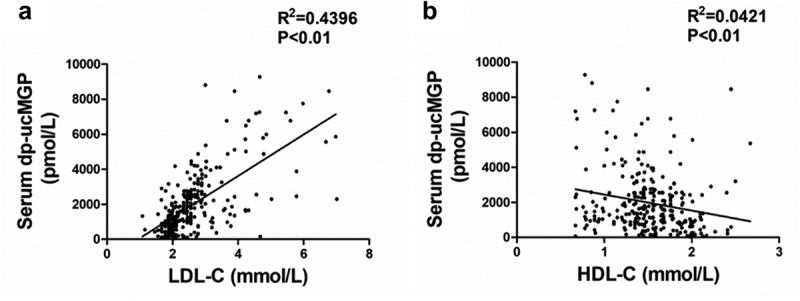
Serum dp-ucMGP positively correlates with LDL-C

Then we analysed the correlation of serum dp-ucMGP levels with lipid metabolism indicators including LDL-C and HDL-C. We found a significant positive correlation between serum dp-ucMGP levels and LDL-C (Figure 4(a)). As shown in Figure 4(b), there is a negative correlation between serum dp-ucMGP levels and HDL-C. These findings demonstrated that circulating dp-ucMGP levels are closely related to lipoprotein level.

(a) The correlation of serum dp-ucMGP levels with LDL-C concentration; (b) The correlation of serum dp-ucMGP levels with HDL-C concentration. n = 278.

## Discussion

Central obesity or abdominal obesity is defined as excess accumulation of fat in abdomen [1]. With the rapid changing of life styles in modern world, more and more people are getting central obesity. It is reported that the prevalence of abdominal obesity in Chinese adults have more than doubled in the last three decades [27]. Abdomen obesity is not readily noticeable but causes serious health problem. It is associated with a higher risk of cardiovascular disease, type 2 diabetes, and other obesity-related disease than general obesity [2,3]. BMI, which is widely used to evaluate general obesity, could not distinguish the regional distribution of adipose tissue [5]. Instead, waist circumference (WC), WHtR, or waist to hip ratio (WHR) are used to define central obesity. These parameters describe the distribution of body fat more accurately and better predict obesity-related health risk than general obesity measured by BMI [28].

In this study, we demonstrated that MGP is highly expressed in fat, especially the visceral fat in mice. MGP expression is significantly induced during preadipocyte 3T3-L1 differentiation. Knockout of MGP leads to retardation of 3T3-L1 differentiation, suggested by the decreased Oil Red staining signal and decreased expression of adipogenetic markers CEBPα and FABP4. Lipid storage indicated by the intracellular triglyceride amount is impaired in MGP-depleted 3T3-L1 cells. The release of glycerol into the cell culture medium which reflected lipolysis is increased in MGP-deficient cells. We analysed the association between serum dp-ucMGP and obesity in a cohort of 278 Chinese Han people. Our results demonstrated that serum dp-ucMGP level is positive associated with visceral fat index (VFI), waist height ratio (WHtR), but not body mass index (BMI). Serum dp-ucMGP positively reflects low-density lipoprotein cholesterol (LDL-C) level. Taken together, our study uncovers the link between MGP function and fat metabolism, sheds light on the potential of dp-ucMGP as a novel serum marker.

Adipose tissue expands in two ways: through differentiation of resident tissue precursors to form new adipocytes (hyperplasia) or enlargement of existing adipocytes (hypertrophy). Adipocyte hyperplasia is generally thought to be healthy, since it is able to maintain proper vascularization and secrete insulin-sensitizing and metabolism-regulatory adipokines [11]. Adipocyte hypertrophy often causes hypoxia because of their massively expanded size, leading to tissue fibrosis, necrosis, immune cells infiltration, and inflammation. It is believed that obesity-associated metabolic problems are not due to adipocyte hyperplasia or adipogenesis, but to adipocyte hypertrophy or adipose dysfunction. Adipose dysfunction results in impaired capacity to store glucose and lipid in adipocytes, subsequent release into blood and accumulation in other tissues (such as muscle and liver), and finally insulin resistance and earlier onset of metabolic disease [11].

PPARγ is characterized as the key transcription factor in adipogenesis, as it is indispensable for adipocyte differentiation [12]. The downstream effects of PPARγ include expression of C/EBPα, FABP4, and adiponectin. In our study, we found that MGP silence impairs the expression of C/EBPα and FABP4, suggesting a stimulatory role of MGP in adipogenesis. These results are consistent with the decreased Oil Red staining signal after MGP was depleted. However, for some unknown reason, the expression of PPARγ and adiponectin remains to be unchanged after MGP was knockout. MGP may play its role in adipogenesis in a PPARγ-independent manner. Further study is needed to identify the downstream pathway. Lipid accumulation and lipolysis are ‘gain’ and ‘loss’ in fat amount maintenance. Our results demonstrated that MGP promotes lipid accumulation while suppressing lipolysis. MGP functions at both directions.

MGP is known as a potent inhibitor of mineralization [14]. Mice deficient in *Mgp* show severe vascular calcification and die in the first weeks of their lives [29]. Increasing evidence shows that circulating uncarboxylated MGP is associated with cardiovascular disease [30]. However, until now there is no study reporting the correlation of MGP with obesity. In the present study, we analysed the association of serum inactive MGP with obesity, and found that dp-ucMGP is positively associated with central obesity. These results are consistent with Mutch’s findings. This association analysis is a cross-sectional study, which is hard to draw conclusion of which one (visceral adipose tissue, MGP function) is the cause and which one is the effects. In addition, 18.7% of the participants have the CVD history, which may have some impact on the circulating dp-ucMGP level. Moreover, we will extend the study to other races or ethnicities. We will further analyse the association between obesity and vitamin K intake.

Our results demonstrated that warfarin-treated mice display increased body fat rate, due to inhibition of MGP. However, Lanham and colleagues reported that *Mgp* knockout mice have lower body weights and less adipose tissue property [20]. This conflict may be caused by several ways: 1) The non-specific inhibition of MGP by warfarin. Warfarin also blocks other vitamin K-dependent proteins. It is possible that other vitamin K-dependent protein compensates the MGP-induced effects; 2) The duration of warfarin treatment. In this study, we treated mice with warfarin for 8 weeks before examination. Although it is long enough to detect the difference, but it is still a temporary interference. For the knockout mice, there is a deletion of MGP in gene level, causing permanent effects. Further evidence is needed to explain fully the conflict.

We demonstrated that MGP regulates adipogenesis in vitro. However, it is difficult to explain why serum dp-ucMGP level is closely associated with visceral adipose. Based on out *in vitro* evidence, MGP inactivation results in increased lipid release out of the adipocyte. This result is consistent with the warfarin mediated inhibition of MGP, as indicated by the impaired Oil Red staining of the gonadal fat tissue section. From this point of view, MGP may inhibit the progression of visceral fat. However, MGP is also expressed in other tissues, such as vasculature, kidneys, lungs [14]. It is possible that serum dp-ucMGP comes from the other tissues. Other function of MGP in these tissues may be involved. More evidence is needed to further clarify the role of MGP in fat metabolism.

Warfarin is an anticoagulant used to reduce the formation of blood clots in veins or arteries, which can reduce the risk of stroke, heart attack, or other serious conditions. Millions of patients have been treated with warfarin for years. However, there is still several limitations and adverse effects. It increases the risk of severe or foetal bleeding. Necrosis, purple toe syndrome, valve and artery calcification, and drug interactions have also been documented with warfarin use [31]. As far as we known, there is no study reporting the effects of warfarin on adiposity or serum lipids until now. Our results indicate another potential side effect of warfarin. It may draw more attention to the abdominal obesity caused by warfarin. Taken all together, our data reveals for the first time that MGP plays important role in fat metabolism, and sheds light on the potential of dp-ucMGP as a novel serum marker for central obesity.

## Supplementary Material

Supplemental Material
