# Supplementary material for: Matrix Gla protein regulates adipogenesis and is serum marker of visceral adiposity
Source: Adipocyte. 2020 Jan 31;9(1):68–76. doi: 10.1080/21623945.2020.1721692 (PMC6999844; doi:10.1080/21623945.2020.1721692)
Supplement: Supplemental Material [file KADI_A_1721692_SM0901.docx]

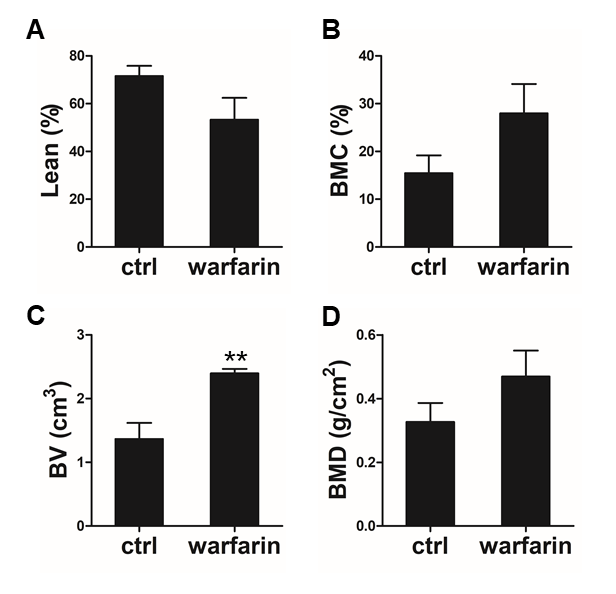


*Figure S1 Effects of warfarin on body composition*

Mice was treated with 300 ng/mL warfarin for 8 weeks, and body composition was measured by DEXA. n=6. A Lean proportion; B Bone mineral content (BMC) proportion; C Bone volume (BV); D Bone mineral density (BMD).
